# Chlorosubstituted Copper Phthalocyanines: Spectral Study and Structure of Thin Films

**DOI:** 10.3390/molecules25071620

**Published:** 2020-04-01

**Authors:** Alexandr Sukhikh, Dmitry Bonegardt, Darya Klyamer, Pavel Krasnov, Tamara Basova

**Affiliations:** 1 Nikolaev Institute of Inorganic Chemistry SB RAS, 3 Lavrentiev Pr., 630090 Novosibirsk, Russia; a_sukhikh@niic.nsc.ru (A.S.); bonegardt@niic.nsc.ru (D.B.); klyamer@niic.nsc.ru (D.K.); 2 Laboratory of Non-Linear Optics and Spectroscopy, Siberian Federal University, 79 Svobodny Prospect, 660041 Krasnoyarsk, Russia; kpo1980@gmail.com

**Keywords:** phthalocyanines, single crystal structure, thin films, X-ray diffraction, vibrational spectra, DFT calculations

## Abstract

In this work, the tetra-, octa- and hexadecachloro-substituted copper phthalocyanines CuPcCl_x_ (where x can equal 4, 8 or 16) were investigated by the methods of vibrational (IR and Raman) spectroscopy and X-ray diffraction. The assignment of the most intense bands, both in IR and Raman spectra, was carried out on the basis of DFT calculations. The structure of a CuPcCl_4_ single crystal grown by sublimation in vacuum was refined for the first time. The effect of chloro-substitution on the structure of CuPcCl_x_ thin films deposited in a vacuum onto a glass substrate at 50 and 200 °C was studied. It was shown that CuPcCl_4_ formed polycrystalline films with the preferential orientation of the (100) crystallographic plane of crystallites parallel to the substrate surface when deposited on a substrate at 50 °C. Introduction of more Cl-substituents into the phthalocyanine macrocycle leads to the formation of amorphous films on the substrates at 50 °C. At the elevated substrate temperature, the growth of polycrystalline disordered films was observed for all three copper phthalocyanines.

## 1. Introduction

Metal phthalocyanines (MPcs) attract the wide attention of researcher in various areas of materials science, chemistry and electronics. For their application in different electronic devices, they should be prepared as thin layers with an easily reproducible structure and ordering. Although halogen-substituted phthalocyanines were synthesized a long time ago [1], the interest in their thin films has increased again in recent years. This is primarily due to their comparatively high charge carriers’ mobility and *n*-type semiconducting behavior [2]. The literature analysis shows that a number of works are devoted to the investigation of fluoro-substituted metal phthalocyanines, as active layers of organic field-effect transistors (OFETs) [3,4,5]. Note that the structural feature and the orientation of molecules relative to the substrate surface in their films have a noticeable effect on the mobility of charge carriers and other OFET characteristics [6,7]. For instance, the mobility of charge carriers in OFETs on the basis of CuPcF_16_ can vary from 10^−3^ [8] to 0.27 cm^2^/V·s [6] in dependence on the structure of films and the dielectric layers. The structure of single crystals and thin films of fluorinated metal phthalocyanines MPcF_x_ (M = Co, Zn, Pd, VO; x = 4, 16) were studied in a series of our previous publications [9,10,11].

The literature analysis shows that the most works on chlorinated metal phthalocyanines are devoted to their synthesis [12,13,14], behavior in solutions [15] and applications as pigments [16,17]. At the same time, the films of chloro-substituted phthalocyanines are also considered as active layers of molecular electronic devices, however such works on the investigation of the structure and properties of their films are sporadic. For example, Pakhomov et al. mentioned that CuPcCl_4_ films can be deposited by vacuum sublimation, but did not characterize them [18]. Several works were devoted to the epitaxial growths of monolayers or ultrathin films on graphite, graphene and KBr (001), and their investigation by the methods of high-resolution electron microscopy [19,20,21,22] and photoelectron spectroscopy [23]. Haruta et al. [24] studied grain boundaries in the thin films of CuPcCl_16_ using an aberration-corrected scanning transmission electron microscope combined with electron energy-loss spectroscopy. Fryer [25] determined the parameters of crystal cell of CuPcCl_x_ (x = 4, 8, 16) using the method of electron diffraction. 

Similarly to unsubstituted and fluoro-substituted derivatives, chloro-substituted metal phthalocyanines exhibit semiconductor properties. According to DFT calculations, the HOMO and LUMO energy levels of CuPcCl_x_ decrease in comparison with unsubstituted CuPc derivatives [26,27]. Koshy et al. [28] investigated the effect of annealing temperature on the optical band gap of FePcCl_16_ films, and considered their possible applications as active layers of molecular electronic devices. Anchar et al. [29] have shown that the conductivity of CuPcCl_4_ pellets is about four orders of magnitude higher than that of unsubstituted CuPc. Ling, Bao and Erk determined that the carrier mobility of a CuPcCl_16_ film deposited at an elevated substrate temperature in air-stable *n*-channel transistors was ~10^−2^ cm^2^ V^−1^ s^−1^ [30]. It is known that the electrical properties of phthalocyanines and other molecular crystals are dependent upon the phase composition, morphology and orientation of molecules in thin films [6,7,28]. It is worth mentioning that in most of the abovementioned articles, the structure of thin films MPcCl_x_ was not studied. For this reason, the study of the structural features of MPcCl_x_ films deposited under different conditions is an important task. In addition to X-ray diffraction techniques, spectral methods, including IR and Raman spectroscopies, are also very informative methods for the investigation of the structural features of phthalocyanine films. For this reason, the detailed assignment of vibrations in IR and Raman spectra is a vital issue.

In this work, the tetra-, octa- and hexadecachloro-substituted copper phthalocyanines CuPcCl_x_ (x = 4, 8, 16) were investigated by the methods of vibrational (IR and Raman) spectroscopy and X-ray diffraction. The assignment of the most intense bands, both in IR and Raman spectra, was carried out on the basis of DFT calculations. The structure of a CuPcCl_4_ single crystal was refined for the first time. The effect of chloro-substitution on the structure of CuPcCl_x_ thin films deposited in vacuum onto the substrate at different temperatures was studied.

## 2. Results and Discussion

### 2.1. Single Crystal Structure of CuPcCl_4_

A single crystal of CuPcCl_4_ in the form of a small needle (0.15 mm × 0.03 mm × 0.03 mm) of dark purple color was selected from a polycrystalline crust formed on the side of a glass ampule during the gradient vacuum sublimation of the product obtained after the synthesis. The unit cell parameters at 150 and 298 K, and the crystal structure refinement statistics are given in Table 1. CuPcCl_4_ crystallizes in a monoclinic crystal system, with a P2_1_/c space group, and *Z* = 2. It is worth mentioning that different unit cell parameters for CuPcCl_4_ were reported by Honigmann et al. [31] (a = 26.3 Å, b = 27.8 Å, c = 27.8 Å, β = 94°, P2_1_/a space group) and Fryer [25] (*a* = 27.4 Å, *b* = 27.4 Å, *c* = 3.65 Å, P4 space group or C2/c space group with β = 90°). Although these unit cell parameters differ significantly from those obtained from a single crystal in this work, this does not necessarily mean that CuPcCl_4_ has several different polymorphs. In the first case, the lattice parameter *c* appears to be doubled (27.8 Å instead of 13.9 Å), and the parameter *b* is multiplied by 8 (27.8 Å instead of 3.48 Å), while the lattice parameter *a* and the monoclinic angle *β* agree fairly well with the single-crystal data. 

In the second case, the unit cell parameters of CuPcCl_4_ were derived from electron diffraction patterns, which have the lower accuracy compared to X-ray diffraction. Thus, the difference between the single-crystal data and the reported unit cell parameters may be caused by instrumental limitations and an incorrect assignment of Miller indices. 

**Figure 1:**
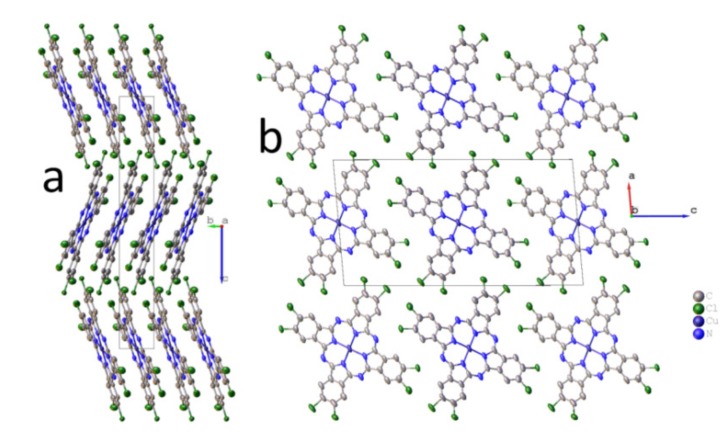
Packing diagrams of CuPcCl_4_ molecules along the a axis (**a**) and b axis (**b**). Chlorine atoms partially occupy eight positions (0.514/0.486 and 0.516/0.484 for Cl1/Cl2 and Cl3/Cl4, respectively).

Packing diagrams of CuPcCl_4_ molecules are shown in Figure 1. CuPcCl_4_ molecules are almost flat (the maximum deviation from the mean squared plane is 0.12 Å for one of the chlorine atoms), being packed in columns along the *b* axis (Figure 1b), and arranged in the herringbone configuration (Figure 1a).

Chlorine atoms partially occupy eight positions (0.514/0.486 and 0.516/0.484 for Cl1/Cl2 and Cl3/Cl4, respectively) because the synthesized CuPcCl_4_ consists of four regioisomers co-crystallizing in one crystal phase. The distance between two molecules (the mean-square plane through all atoms in the molecule, except hydrogen) in a stack is 3.376 Å, while the stacking angle (the angle between the normal to the plane of the molecule and b-axis) is 21.88° (27° as reported in Fryers work [25]). The angle between two molecules in adjacent stacks is 43.76°. These values differ significantly from those reported for a stable β-polymorph of unsubstituted copper phthalocyanine (3.342 Å and 45.76°) [32], in which molecules in adjacent stacks are oriented almost perpendicularly to each other, and for CuPcF_4_ [11] (3.381 Å and 24.29°), in which molecules in adjacent stacks are parallel to each other. A similar stacking pattern, however, is observed in γ-CuPc (3.386 Å and 27.37°) [33]. Although γ-CuPc crystallizes in the other space group (C2/c), its unit cell parameters (a = 26.3330 Å, b = 3.8133 Å, c = 23.7118 Å, β = 94.2845°) are similar to CuPcCl_4_.

**Figure 2:**
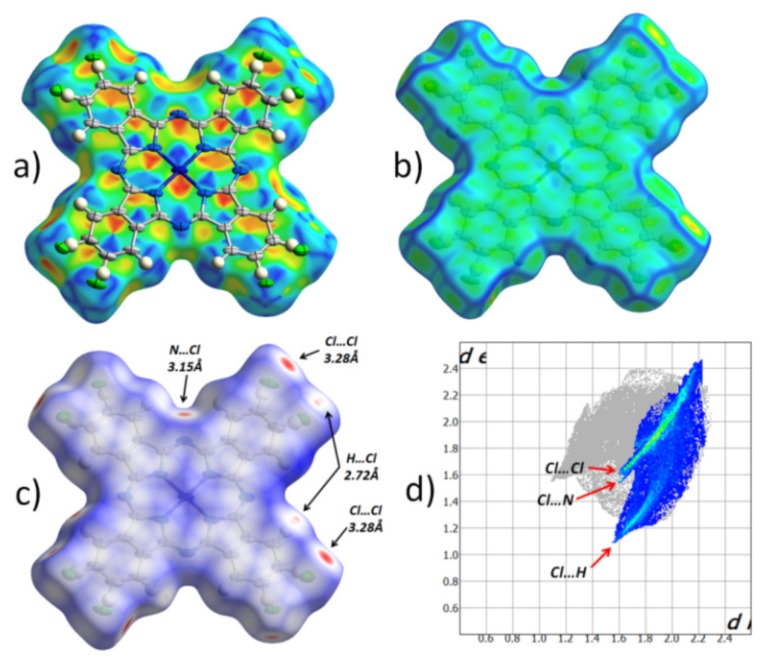
Hirshfeld surface for CuPcCl_4_ molecule, mapped with the shape index (**a**), curvedness (**b**), normalized contact distance (d_norm_, −0.15… + 1 range) (**c**) and the fingerprint plot for all intermolecular contacts with chlorine atoms (**d**).

Figure 2 shows the Hirshfeld surface for an individual CuPcCl_4_ molecule, mapped with different properties (shape index, curvedness and normalized contact distance), as well as the fingerprint plot for all intermolecular contacts with chlorine atoms that occupy 32.4% of the entire Hirshfeld surface. The Hirshfeld surface mapped with the shape index (Figure 2a) is shown with the adjacent molecule in the stack, superimposed over it.

Pairs of red and blue triangles (Figure 2a), located in the shape of an hourglass, are visible inside each of the benzene and pyrrole rings, as well as in all four quadrants of the central phthalocyanine macrocycle. These features together with the uniform flat surface (Figure 2b) clearly show π‒π interaction between CuPcCl_4_ molecules in one stack. Moreover, each individual aromatic segment of one molecule interacts with the same segment of a neighboring molecule. The same type of π‒π interaction was previously observed in CuPcF_4_, but not in β-CuPc [11]. The Hirshfeld surface mapped with the normalized contact distance (Figure 2c; d_norm_, −0.15… + 1 range) shows that there are no other close contacts between the molecules in one stack. However, unlike CuPcF_4_ and β-CuPc, which have almost no close contacts, there are several close contacts between CuPcCl_4_ molecules in adjacent stacks, and they all involve peripheral chlorine atoms. The fingerprint plot (Figure 2d) shows three distinct groups of close contacts, viz. 3.28 Å Cl..Cl, 3.15 Å Cl..N_β_, 2.72 Å Cl..H. 

The most prominent close contacts are Cl..Cl ones, which are ~0.2 Å shorter than the sum of van der Waals radii of two chlorine atoms. However, these contacts are not necessarily present in each individual CuPcCl_4_ molecule, because all atomic positions of chlorine are only partially occupied. This may also explain the strange appearance of thermal ellipsoids (Figure 2a), such that they are all elongated in the same direction. It is possible that, depending on the presence of chlorine atoms in certain positions associated with Cl..Cl close contacts, CuPcCl_4_ molecules can shift slightly inside the stack to minimize the stress caused by close contacts.

### 2.2. Vibrational Spectra

**Figure 3:**
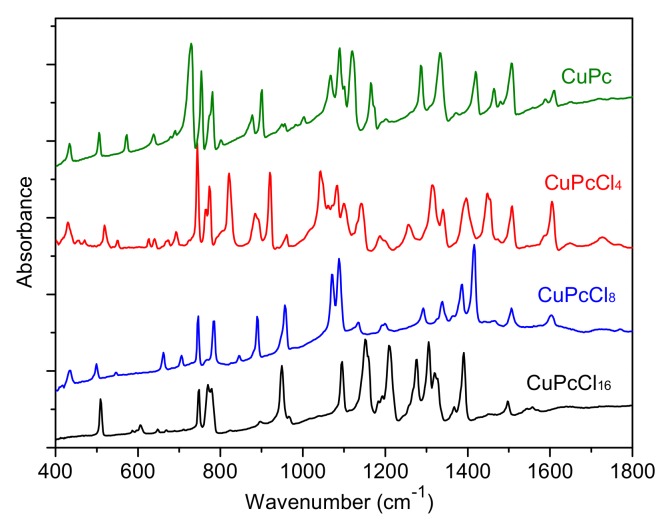
Infrared (IR) spectra of CuPcCl_x_ (x = 0, 4, 8, 16).

**Figure 4:**
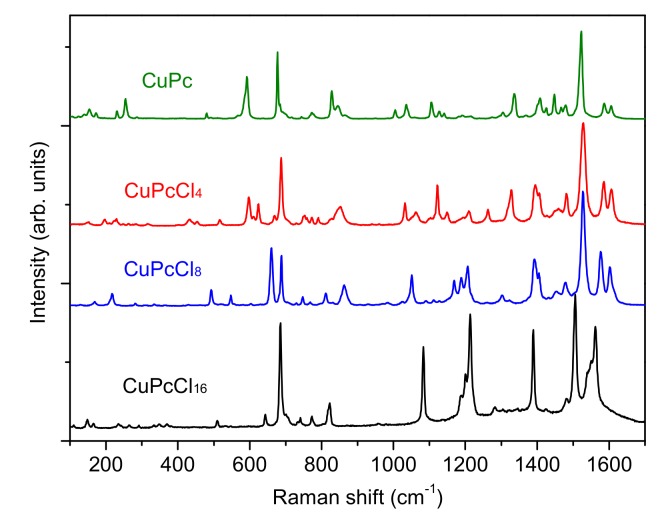
Raman spectra of CuPcCl_x_ (x = 0, 4, 8, 16).

**Figure 5:**
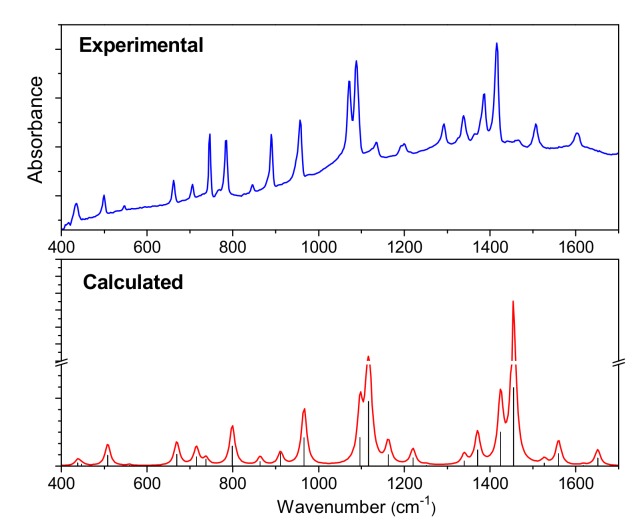
Experimental and calculated IR spectra of CuPcCl_4_.

The IR and Raman spectra of CuPcCl_x_ (x = 4, 8, 16) are given in Figure 3 and Figure 4, respectively. The spectra of unsubstituted CuPc are also given for comparison. The assignment of vibrational spectra was based on the results of quantum chemical calculations. The calculated spectra are in good agreement with the experimental ones. A comparison of the calculated and experimental IR spectrum of CuPcCl_4_ is presented in Figure 5 as an example. Tables S1–S4 (Supplementary Materials) summarize the experimental and calculated wavenumbers, as well as the assignment of the most intense bands in IR and Raman spectra of CuPcCl_x_ derivatives. 

CuPcCl_4_ and CuPcCl_8_ are planar molecules of D_4h_ and C_4h_ point group symmetry, while the introduction of 16 chlorine substituents into the phthalocyanine macrocycles leads to a non-planar distortion of the CuPcCl_16_ macrocycle, and as a result, a decrease of the molecule symmetry to D_2d_. 

A comparison of experimental and DFT-optimized bond lengths in CuPcCl_x_ molecules is given in Table 2. 

The introduction of bulky Cl-substituents to the phthalocyanine macrocycle leads to the shift of some modes in the range from 1350 to 1620 cm^−1^ to the lower wavenumbers compared to CuPc [34]. These modes are attributed to C=С and C=N stretching vibrations and isoindole deformations. For example, the mode at 1608 cm^−1^ in the IR spectrum of CuPc attributed to C=С stretchings shifts to 1605, 1602 and 1558 cm^−1^ for CuPcCl_4_, CuPcCl_8_ and CuPcCl_16_, respectively, due to the induction (-I) effects of electron-withdrawing Cl-atoms. The introduction of Cl-substituents causes the change of both the intensity and wavenumbers of the most bands in the range from 700 to 1300 cm^−1^ because of the change of the forms of the corresponding vibrations and the strong contributions of C–Cl vibrations. This is especially noticeable in the case of CuPcCl_8_ and CuPcCl_16_ derivatives. Even the group of bands at 500−900 cm^−1^, where the most vibrations are attributed to macro-ring deformations along with Cu-N_α_ stretchings, is not left unchanged.

Similar changes are also observed in the Raman spectra. The spectra of CuPc and CuPcCl_4_ are similar, whereas the introduction of 8 and 16 chlorine substituents causes noticeable shifts and the change of intensities of most vibrations. The bands belonging to C=С stretching vibrations at 1607 and 1589 cm^−1^ observed in the case of CuPc shift to the lower wavenumbers in the Raman spectra of CuPcCl_4_, CuPcCl_8_ and CuPcCl_16_. The strong bands at 1528 cm^−1^ in the spectra of CuPcCl_4_ and CuPcCl_8_, corresponding to C_α_-N_β_ and C_β_-C_β_ stretchings, are not shifted relative to the corresponding band in the spectrum of CuPc, while in the spectrum of CuPcCl_16_, this band is observed at 1506 cm^−1^. When compared with the spectra of CuPc and CuPcCl_4_, the intensities of bands at 492, 660 and 1050 cm^−1^ in the CuPcCl_8_ spectrum, and at 643 and 1078 cm^−1^ in the CuPcCl_16_ spectrum, increase due to the contribution of C–Cl stretchings to these vibrations. It is interesting to mention that the totally symmetric bands with dominating contribution of C_α_–N_β_–C_α_ deformation do not practically change and locate at 689, 688 and 685 cm^−1^ in the spectra of CuPcCl_4_, CuPcCl_8_ and CuPcCl_16_. At the same time the wavenumbers of totally symmetric bands located at 820–850 cm^−1^ vary because of different forms of these vibrations: in the Raman spectra of CuPcCl_4_ and CuPcCl_8_, these vibrations at 828 and 832 cm^−1^ are associated with macro-ring breathing with the contribution of C_β_–C_γ_–H, while in the case of CuPcCl_16_, this mode lies at 821 cm^−1^, and involves not only macro-ring and C_β_–C_γ_–H deformations, but also C_γ_–Cl and C_δ_–Cl.

### 2.3. XRD Study of CuPcCl_4_, CuPcCl_8_ and CuPcCl_16_ Thin Films

**Figure 6:**
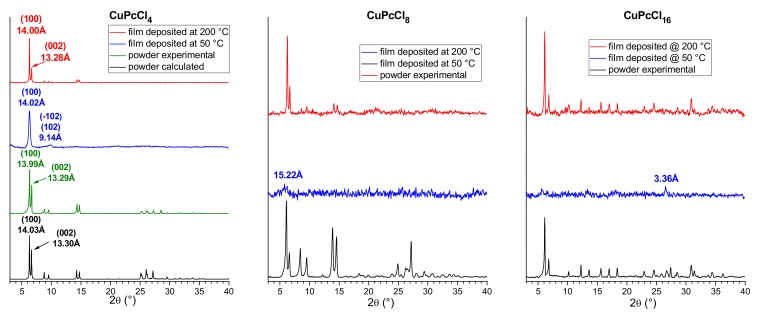
Diffraction patterns of CuPcCl_4_, CuPcCl_8_ and CuPcCl_16_ polycrystalline powders and thin films deposited at 50 °C and 200 °C.

Thin films of CuPcCl_4_, CuPcCl_8_ and CuPcCl_16_ were deposited onto glass substrates at two different temperatures 50 and 200 °C. The diffraction patterns for CuPcCl_4_, CuPcCl_8_ and CuPcCl_16_ polycrystalline powders and thin films are shown in Figure 6.

**Figure 7:**
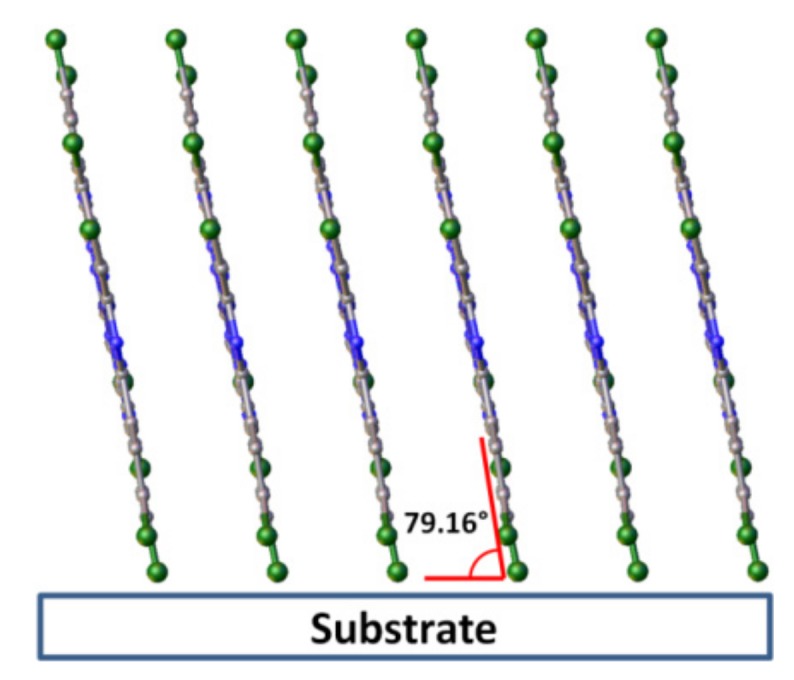
Orientation of CuPcCl_4_ molecules relative to the substrate surface in thin films.

CuPcCl_4_ powder was compared with the diffraction pattern calculated using single-crystal data obtained at 150 K and unit cell parameters measured from the same single crystal at room temperature (298 K). The experimental peak positions and intensities match well with the calculated ones, and no additional diffraction peaks are observed, indicating that the CuPcCl_4_ powder is single phase. A single strong diffraction peak is observed on the diffraction pattern of CuPcCl_4_ thin film deposited onto a substrate at 50 °C. Since the 2θ position of this peak almost perfectly coincides with the (100) peak in the calculated diffraction pattern of the powder, it is possible conclude that this thin film consists of the same crystalline phase as the bulk polycrystalline powder, but has a strong preferred orientation with the (100) crystallographic plane of the majority of crystallites oriented parallel to the substrate surface. The inclination angle of CuPcCl_4_ molecules relative to the substrate surface (Figure 7) was estimated to be 79.2° (cf. 76.6° for CuPc and 82.7 for CuPcF_4_). In addition to the strong (100) peak, the diffraction pattern shows a weak and blurred diffraction peak with the interplane distance of 9.14 Å, which can be attributed to the planes (−102) and (102), which means that the preferred orientation of CuPcCl_4_ is not ideal.

Multiple diffraction peaks are observed on the diffraction pattern of a CuPcCl_4_ film deposited on the substrate at 200 °C, indicating that it has no preferred orientation (Figure 6). 2θ positions of all observed peaks also coincide well with the calculated pattern of the powder. This means that CuPcCl_4_ films consist of the same crystal phase, and there is no evidence of other polymorphic modifications. CuPcCl_8_ and CuPcCl_16_ thin films deposited on the substrates at 50 °C are amorphous (Figure 6). Their diffraction pattern contains almost no diffraction peaks, except for very weak peaks at 15.22 Å for CuPcCl_8_, and at 3.36 Å for CuPcCl_16_. On the other hand, the films deposited on the substrates at 200 °C are crystalline, and multiple diffraction peaks are observed on their diffraction patterns. 2θ positions of the diffraction peaks of these films coincide with the corresponding powder diffraction patterns, which means that the CuPcCl_8_ and CuPcCl_16_ films deposited at 200 °C have the same phase composition as their polycrystalline powders.

The unit cell parameters measured using electron microdiffraction [35] are available in the literature for CuPcCl_16_: *a* = 19.62 Å, *b* = 26.04 Å, *c* = 3.76 Å, β = 116.5°, C2/c or C2/m space group. Based on these data, the CuPcCl_16_ diffraction pattern was indexed, and the unit cell parameters were refined using the 2θ positions of the diffraction peaks measured from the powder diffraction pattern (see Table S6). The refined unit cell parameters for CuPcCl_16_ are: a = 19.16(2) Å, b = 26.03(3) Å, C = 3.695(3) Å, β = 114.55(1)°, V = 1675(3) Å^3^. All diffraction peaks on the CuPcCl_16_ XRD pattern are indexed within the specified unit cell parameters, which means that both the film deposited on the substrate at 200 °C and the bulk powder consist of one crystal phase. The only available XRD data for CuPcCl_8_ are the 2θ positions of several strongest diffraction peaks measured from the powder diffraction pattern (6.1°, 6.6°, 8.5°, 9.5°, 14.0°, 14.5°, 24.9° and 27.1°, CuKα radiation) [36], which match well with the strongest peaks observed in our XRD data. Therefore, the appropriate unit cell parameters for CuPcCl_8_ were found using the DICVOL14 indexing program [37]. Since V/Z ratios (volume of one molecule) of CuPcCl_4_ and CuPcCl_16_ are known (689.7 and 859.6 Å^3^, respectively), they were used as the lower and upper limits for the volume of the CuPcCl_8_ molecule in the search process. The obtained unit cell parameters for CuPcCl_8_ are: *a* = 8.01(1) Å, b = 13.64(2) Å, c = 14.75(2) Å, α = 82.03(7)°, β = 82.03(9)°, γ = 80.59(8)°, V = 1564(3) Å^3^, P−1 space group. All diffraction peaks on the CuPcCl_8_ XRD pattern with their 2θ positions, intensities and assigned hkl indices are listed in Table S7. It is worth mentioning that another octa-halogenated phthalocyanine, ZnPcF_8_, has similar unit cell parameters (a = 8.196 Å, b = 11.493 Å, c = 13.995 Å, α = 78.460°, β = 73.501°, γ = 89.078°) [38], determined from single crystal data. This may serve as indirect confirmation that the indexing was correct, and that CuPcCl_8_ may have a molecular packaging style similar to ZnPcF_8_.

## 3. Materials and Methods

CuPcCl_x_ derivatives were synthesized by the method of template synthesis from the corresponding chloro-substituted phthalonitriles (Sigma Aldrich, Saint Louis, USA) and CuCl [39]. For purification the obtained powders were sublimed in vacuum (10^−5^ Torr) twice. The sublimation temperatures were 430, 460 and 490 °C for CuPcCl_4_, CuPcCl_8_ and CuPcCl_16_, respectively. Thin films of CuPcCl_x_ (x = 4, 8, 16) were deposited by physical vapor deposition onto glass substrates at two different substrate temperatures of 50 and 200 °C. The nominal thickness of the deposited films varied in the range of 100–150 nm. 

IR spectra of CuPcCl_x_ in KBr pellets were recorded with a Vertex 80 FTIR spectrometer (Ettlingen, Germany) while those Raman spectra of the CuPcCl_x_ powders were measured with a LabRAM Horiba single spectrometer (HORIBA, Montpellier, France) (488 nm line of an Ar+ laser). 

The crystal structure of CuPcCl_4_ was determined using a Bruker X8 single-crystal diffractometer (Billerica, MA, US) (a MoKα sealed tube with a graphite monochromator and Apex II CCD detector). The temperature of crystal was kept at 150 K by an Oxford Cryosystems Cryostream 800 (Oxford, United Kingdom) plus open-flow nitrogen gas cooler. Data collection strategy consisted of conventional φ- and ω-scans with 0.5° wide frames. The Apex3 v.2018-7.2 (Madison, Wisconsin, USA) software package [40] was used for data reduction and absorption correction. The crystal structure was processed in Olex2 v.1.2.10 (Durham, United Kingdom) [41] with SHELXT−2018/2 Göttingen, Germany) [42] and SHELXL−2018/3 (Göttingen, Germany) [43] used for the initial structure solution and subsequent refinement, respectively. Additionally, the unit cell parameters of the same crystal were measured at 298 K using 119 individual reflections. Hirshfeld surfaces mapped with different properties (curvedness, shape index, normalized contact distance) were generated in CrystalExplorer 17.5 (Perth, Western Australia, Australia) [44] using a Tonto v.17.04 computational chemistry package (Perth, Western Australia, Australia) [45]. Diffraction patterns of bulk polycrystalline powders and thin films were recorded on a Shimadzu XRD−7000 powder diffractometer (Kyoto, Japan) (a CuKα sealed tube with Ni β-filter, Bragg-Brentano geometry with a vertical θ-θ goniometer and OneSight SSD detector). 

Quantum-chemical calculations of geometries and IR and Raman spectra of CuPcCl_x_ (x = 4, 8, 16) molecules were carried out by the DFT B3LYP/6–31G(d,p) method [46,47,48,49] in the frame of spin-unrestricted Kohn–Sham theory (UKS) with use of GAMESS software (version 20.04.2017 (R1), Ames, IA, USA) [50]. Doublet spin states only were considered in all three cases. Geometry relaxation of the CuPcCl_4_, CuPcCl_8_ and CuPcCl_16_ molecules was constrained by their corresponding symmetries, such as C_4h_, D_4h_ and D_2d_.

## 4. Conclusions

In this work, tetra-, octa- and hexadecachloro-substituted copper phthalocyanines CuPcCl_x_ (x = 4, 8, 16) were investigated by the methods of vibrational (IR and Raman) spectroscopy and X-ray diffraction. The assignment of the most intense bands both in IR and Raman spectra was carried out on the basis of DFT calculations. It was shown that CuPcCl_4_ and CuPcCl_8_ were planar molecules of D_4h_ and C_4h_ point group symmetry, while the introduction of 16 chlorine substituents into the phthalocyanine macrocycles led to a non-planar distortion of the CuPcCl_16_ macrocycle, and as a result, a decrease of the molecule symmetry to D_2d_. The introduction of Cl-substituents led to the change of both intensities and forms of most vibrations in IR and Raman spectra. 

The structure of a CuPcCl_4_ single crystal grown by sublimation in vacuum was refined for the first time. CuPcCl_4_ was shown to crystallize in the monoclinic P2_1_/c space group with the following unit cell parameters at 150 K: Z = 2, a = 14.0052(9) Å, b = 3.6376(3) Å, c = 26.5123(18) Å, β = 94.893(3)°. 

The effect of chloro-substitution on the structure of CuPcCl_x_ thin films deposited in vacuum onto the substrate at 50 and 200 °C was studied. It was shown that when deposited on a substrate at 50 ^°^C, CuPcCl_4_ formed polycrystalline films with the preferential orientation of the (100) crystallographic plane of crystallites parallel to the substrate surface and the inclination angle of molecules relative to the substrate surface of 79.2°. Introduction of more Cl-substituents into the phthalocyanine macrocycle led to the formation of amorphous films on the substrates at 50 °C. At elevated substrate temperatures, the growth of polycrystalline disordered films was observed for all three CuPcCl_x_ (x = 4, 8, 16) phthalocyanines. These data can be used for the interpretation of correlations between the structure and properties of films when creating active layers of electronic devices based on chlorinated metal phthalocyanines. 

## Figures and Tables

**Table 1:** Unit cell parameters and refinement statistics for CuPcCl_4_.

Empirical Formula	C_32_H_12_Cl_4_CuN_8_	
Formula weight	713.84	
Temperature/K	150	298
Crystal system	monoclinic	monoclinic
Space group	P2_1_/c	P2_1_/c
a/Å	14.0052(9)	14.080(4)
b/Å	3.6376(3)	3.6823(8)
c/Å	26.5123(18)	26.693(5)
α/°	90	90
β/°	94.893(3)	94.636(20)
γ/°	90	90
Volume/Å^3^	1345.76(17)	1379.4(5)
Z	2	2
ρ_calc_g/cm^3^	1.762	1.806
Reflections collected	12242	N/A
Independent reflections	2580 (R_int_ = 9.05%)	N/A
Data/restraints/parameters	2508/0/225	N/A
Goodness-of-fit on F^2^	1.010	N/A
R indices [I > = 2σ (I)]	R_1_ = 4.90%, wR_2_ = 9.02%	N/A
R indices [all data]	R_1_ = 12.03%, wR_2_ = 11.05%	N/A
CCDC №	1972791	N/A

**Table 2:** Bond lengths (Å) in CuPcCl_x_ (x = 4, 8, 16) molecules.

Bond	CuPcCl_4_		CuPcCl_8_	CuPcCl_16_
	Experimental	Calculated		
Cu-N_α_	1.942	1.951	1.951	1.950
C_α_-N_α_	1.372	1.377	1.376	1.372
C_α_-N_β_	1.332	1.324	1.324	1.322
C_α_-C_β_	1.454	1.456	1.457	1.465
C_β_-C_β_	1.392	1.405	1.404	1.412
C_β_-C_γ_	1.391	1.396	1.392	1.400
C_γ_-C_δ_	1.376	1.392	1.394	1.404
C_δ_-C_δ_	1.393	1.408	1.418	1.415
C_γ_-H	n/a	1.084	1.084	-
C_δ_-H	n/a	1.084	-	-
C_γ_-Cl	-	-	-	1.736
C_δ_-Cl	1.682	1.758	1.746	1.732

## Supplementary Materials

The following are available online, Table S1: Experimental and calculated IR wavenumbers (cm^−1^) and assignments of the most intense vibrations in the IR spectrum of CuPcCl_4_, Table S2: Experimental and calculated Raman shifts (cm^−1^) and assignments of the most intense vibrations in the Raman spectrum of CuPcCl_4_, Table S3: Experimental and calculated IR wavenumbers (cm^−1^) and assignments of the most intense vibrations in the IR spectrum of CuPcCl_8_, Table S4: Experimental and calculated Raman shifts (cm^−1^) and assignments of the most intense vibrations in the Raman spectrum of CuPcCl_8_, Table S5: Experimental and calculated Raman and IR wavenumbers (cm^−1^) and assignments of the most intense vibrations in the IR and Raman spectrum of CuPcCl_16_, Table S6: Measured peak positions, intensities and assigned hkl indexes for the XRD pattern of CuPcCl_16_, Table S7: Measured peak positions, intensities and assigned hkl indexes for the XRD pattern of CuPcCl_8_.
