# Supplementary material for: Chlorosubstituted Copper Phthalocyanines: Spectral Study and Structure of Thin Films
Source: Molecules. 2020 Apr 1;25(7):1620. doi: 10.3390/molecules25071620 (PMC7181266; doi:10.3390/molecules25071620)
Supplement: Supplementary file 1 [file molecules-25-01620-s001.pdf]

SUPPLEMENTARY MATERIALS

Chlorosubstituted copper phthalocyanines: spectral study and structure of thin films

A. Sukhikh, D. Klyamer, D. Bonegardt, P. Krasnov, and T. Basova

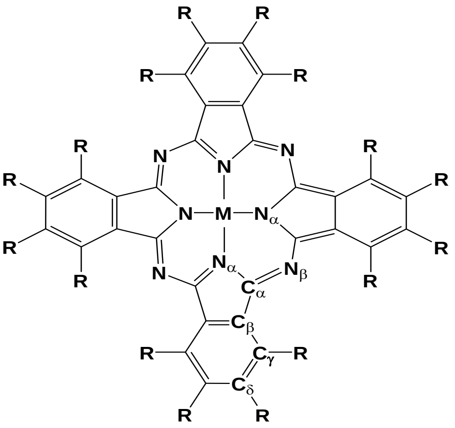

Table S1. Experimental and calculated IR wavenumbers (cm^{-1}) and assignments of the most intense vibrations in the IR spectrum of $CuPcCl_4$.

Experimental	Calculated			Assignments
Wawenumber	Wawenumber	Intensity	Symmetry	
430m	432	0.89	E_u	N_α-Cu, C_γ-C_δ-C_δ, C_γ-C_δ-Cl
454	433	0.17	A_u	C_γ-C_δ-H OOP
470	462	0.16	A_u	C_α, C_β, C_δ OOP motions
521w	516	0.26	E_u	N_α-Cu-N_α, C_α-C_β-C_γ, C_γ-C_δ-Cl
627w	605	0.06	E_u	N_α-Cu, C_β-C_γ-C_δ
640w	615	0.30	A_u	C_γ-C_δ-C_δ, C_γ-C_δ-H OOP
673w	680	1.34	E_u	N_α-Cu, C_β-C_β-C_γ, C_γ-C_δ-C_δ, C_δ-Cl
692w	718	1.26	A_u	N_α-C_α-N_β, C_β-C_γ-H, C_γ-C_δ-H OOP
746	757	0.02	A_u	C_α-C_β-C_γ, C_β-C_γ-H, C_γ-C_δ-H OOP
773s	759	2.41	E_u	N_α-Cu, C_α-N_α-C_α, C_γ-C_δ-C_δ
808sh	818	1.96	A_u	C_β-C_γ-H OOP
822s	819	1.15	E_u	N_α-Cu, C_α-N_β-C_α, C_γ-C_δ-C_δ, C_β-C_γ-C_δ, C_δ-Cl
885m	883	0.71	A_u	C_β-C_γ-H IP
920s	909	4.19	E_u	macroring def., C_δ-Cl
961	946	0.01	A_u	C_β-C_γ-H, C_δ-C_δ-H OOP
1044s	1038	4.75	E_u	C_β-C_γ-H, C_δ-C_δ-H, C_γ-C_δ-C_δ, C_δ-Cl
1084s	1072	2.30	E_u	C_β-C_γ-H, C_δ-C_δ-H, C_δ-C_γ-H, benzene def.
1099s	1105	2.36	E_u	N_α-Cu-N_α, C_β-C_γ-H, C_δ-C_δ-H, C_α-N_α
1142s	1133	3.04	E_u	C_β-C_γ-H, C_δ-C_δ-H, C_δ-C_γ-H, C_α-N_α
1188w	1186	1.05	E_u	C_β-C_β-C_γ, C_β-C_γ-H, C_α-N_α-C_α
1256m	1248	0.83	E_u	C_β-C_γ-H, C_γ-C_δ-H, C_α-C_β-C_β
1315	1325	2.30	E_u	C_α-N_α-C_α, C_α-C_β-C_β, C_δ-C_δ, C_γ-C_δ, C_γ-C_δ-H
1339	1336	3.62	E_u	C_β-C_β, C_δ-C_δ, isoindole def.
1397s	1398	5.62	E_u	Isoindole def., C_β-C_γ-H, C_α-C_β
1449s	1443	3.73	E_u	Benzene def., C_γ-C_δ-H, C_δ-C_δ-H, C_α-C_β
1472	1482	0.42	E_u	C_α-N_β, C_α-C_β, C_β-C_γ-H, C_γ-C_δ-H
1508s	1516	1.89	E_u	C_α-C_β, C_α-N_β
1587sh	1581	0.21	E_u	C_β-C_β, C_δ-C_δ, C_γ-C_δ-H IP
1605s	1605	3.00	E_u	C_α-C_β, C_β-C_γ, C_γ-C_δ

Table S2. Experimental and calculated Raman shifts (cm^{-1}) and assignments of the most intense vibrations in the Raman spectrum of $CuPcCl_4$.

Experimental	Calculated		Assignments
Raman shift	Raman shift	Symmetry	
223	206	A_g	Cu-N_{α}, C_{α}-C_{β}-C_{γ}
230	228	B_g	N_{α}-Cu-N_{α}
263	266	B_g	C_{γ}-C_{δ}-Cl, N_{α}-Cu-N_{α}
285	268	E_g	N_{α}, N_{β} OOP motions, C_{γ}-C_{δ}-H OOP
317	312	A_g	C_{γ}-C_{δ}-Cl, C_{α}-C_{β}-C_{γ}
433	424	A_g	macroring breathing, Cu-N_{α}, C_{γ}-C_{δ}-Cl
454	425	E_g	C_{β}-C_{γ}-H
517	508	B_g	C_{α}-C_{β}-C_{γ}, C_{α}-N_{α}-C_{α}, C_{γ}-C_{δ}-Cl
598	588	B_g	macroring breathing, Cu-N_{α}
612	592	A_g	macroring breathing, Cu-N_{α}
625	619	A_g	macroring def., Cu-N_{α}
670	660	A_g	C_{β}-C_{γ}-C_{δ}, isoindole def., C_{δ}-Cl
688	684	A_g	C_{α}-N_{β}-C_{α}, C_{β}-C_{γ}-C_{δ}
731	711	E_g	N_{α}, N_{β}, C_{α} OOP motions
749	740	B_g	Pyrrole def., Cu-N_{α}
753	745	B_g	macroring breathing
760	755	E_g	macroring def., C_{δ}-Cl
774	794	B_g	macroring breathing, C_{δ}-Cl, Cu-N_{α}
791	817	E_g	C_{β}-C_{γ}-H, C_{γ}-C_{δ}-H OOP
828	837	A_g	macroring breathing, C_{β}-C_{γ}-H
852	883	A_g	Macroring def., C_{δ}-Cl
1033	1029	B_g	C_{β}-C_{γ}-H, C_{α}-N_{β}-C_{α}, benzene def.
1053	1043	A_g	C_{β}-C_{γ}-H, C_{δ}-C_{δ}-H, C_{δ}-Cl
1064	1054	B_g	C_{β}-C_{γ}-H, C_{δ}-Cl, C_{α}-N_{β}-C_{α}
1103	1100	A_g	C_{β}-C_{γ}-H, C_{δ}-Cl, C_{β}-C_{γ}-C_{δ}
1123	1140	A_g	C_{α}-N_{β}-C_{α}, isoindole def., C_{δ}-Cl
1150	1170	B_g	C_{α}-N_{β}-C_{α}, isoindole def., C_{δ}-Cl
1195	1182	A_g	C_{β}-C_{γ}-H, C_{γ}-C_{δ}-H, C_{α}-N_{α}
1210	1202	B_g	C_{β}-C_{γ}-H, C_{α}-N_{α}
1264	1249	B_g	C_{β}-C_{γ}-H, C_{γ}-C_{δ}-H, pyrole def.
1329	1334<br>1335	A_g<br>B_g	C_{β}-C_{β}, C_{δ}-C_{δ}, C_{α}-N_{β}-C_{α}, C_{γ}-C_{δ}-H
1396	1387	A_g	isoindole def., C_{α}-N_{α}, C_{α}-C_{β}
1407	1418	A_g	C_{β}-C_{γ}-H, C_{α}-N_{α}, C_{β}-C_{γ}
1427	1424	B_g	C_{β}-C_{γ}-H, C_{α}-N_{β}, C_{β}-C_{γ}
1482	1498	A_g	C_{α}-N_{β}
1508	1527	A_g	C_{α}-N_{β}, C_{α}-C_{β}, C_{β}-C_{γ}-H
1529	1564	B_g	C_{α}-N_{β}, C_{β}-C_{β}
1587	1583<br>1584	B_g<br>A_g	C_{α}-N_{β}, C_{β}-C_{β}, C_{δ}-C_{δ}<br>C_{β}-C_{β}, C_{δ}-C_{δ}
1607	1603<br>1607	A_g<br>B_g	C_{α}-C_{β}, C_{β}-C_{γ}, C_{γ}-C_{δ}<br>C_{α}-C_{β}, C_{β}-C_{γ}, C_{γ}-C_{δ}

Table S3. Experimental and calculated IR wavenumbers (cm^{-1}) and assignments of the most intense vibrations in the IR spectrum of $CuPcCl_8$.

Experimental	Calculated			Assignments
Wavenumber	Wavenumber	Intensity	Symmetry	
434m	425	0.46	E_u	N_α-Cu-N_α, C_δ-C_δ-Cl, N_α-C_α-N_β
500m	493	1.61	E_u	Cu-N_α, benzene def., C_α-N_β-C_α, N_β-C_α-C_β, C_δ-Cl
546w	542	0.08	E_u	isoindole in-plane def.
662w	649	1.74	E_u	C_γ-C_δ-Cl, C_β-C_γ-C_δ, C_α-C_β-C_γ, C_δ-Cl
706w	694	1.35	E_u	Cu-N_α, pyrole def., C_γ-C_β-C_β, C_δ-Cl, C_α-N_β-C_α
746s	715	1.04	A_{2u}	C_β-C_γ-H OOP, N_β, N_α, C_α OOP motions
785s	776	2.95	E_u	Cu-N_α, C_α-N_α-C_α, C_γ-C_δ-C_δ
845w	838	0.63	E_u	Cu-N_α, C_α-N_β-C_α, C_γ-C_δ-C_δ, C_β-C_γ-C_δ, C_δ-Cl
889m	884	1.93	A_{2u}	C_δ-C_γ-H OOP
957s	937	4.19	E_u	C_α-N_β-C_α, C_γ-C_δ-C_δ, C_δ-Cl
1070s	1064	4.23	E_u	Isoindole def., C_β-C_γ-H, N_α-Cu-N_α
1088s	1083	9.54	E_u	N_α-Cu-N_α, C_β-C_γ-H, C_α-N_α, benzene def.
1134m	1128	1.66	E_u	C_α-N_α, C_β-C_γ-C_δ, C_δ-Cl, C_γ-Cl, C_α-N_β, benzene breathing
1200m	1184	1.19	E_u	C_α-N_α-C_α, isoindole def., C_δ-Cl, C_γ-Cl, benzene breathing
1209sh	1213	0.06	E_u	C_β-C_γ-H, C_γ-C_δ-Cl
1292m	1299	0.76	E_u	C_α-C_β-C_β, C_β-C_β, C_δ-C_δ, C_γ-C_δ-Cl
1339m	1330	2.38	E_u	C_α-N_α-C_α, C_α-N_β-C_α, C_β-C_β, C_δ-C_δ
1387s	1382	5.00	E_u	isoindole def., C_β-C_γ-H, C_α-N_β
1416s	1411	11.59	E_u	benzene def., C_β-C_γ-H, C_α-C_β, C_α-N_β-C_α
1466w	1480	0.41	E_u	N_β-C_α, C_δ-C_γ-H, C_α-C_β
1506m	1513	1.81	E_u	N_β-C_α, C_β-C_γ-H, C_α-C_β
1560w	1568	0.09	E_u	C_β-C_β, C_δ-C_δ, C_γ-C_δ-Cl
1602m	1602	1.18	E_u	C_α-C_β, C_δ-C_γ, C_δ-C_γ-H

Table S4. Experimental and calculated Raman shifts (cm^{-1}) and assignments of the most intense vibrations in the Raman spectrum of $CuPcCl_8$.

Experimental	Calculated		Assignments
Wavenumber	Wavenumber	Symmetry	
218	212	A_{1g}	Cu-N_α, C_γ-C_δ-Cl
281	275	B_{2g}	C_γ-C_δ-Cl, N_α-Cu-N_α
308	299	E_g	N_α, N_β OOP motions, C_γ-C_δ-H OOP
335	339	E_g	C_β and C_δ OOP motions
429	436	B_{2g}	benzene def., C_δ-Cl, C_γ-C_δ-C_δ
492	486	A_{1g}	macroring breathing, Cu-N_α, C_δ-Cl
520	543	B_{2g}	C_α-C_β-C_γ, C_γ-C_δ-Cl, N_α-Cu-N_α
547	545	E_g	N_β, C_α, C_β OOP motions
603	644	B_{1g}	macroring def., Cu-N_α
660	650	A_{1g}	macroring breathing, C_δ-Cl, Cu-N_α
688	684	A_{1g}	C_α-N_β-C_α, C_β-C_γ-C_δ
729	708	E_g	N_α, N_β, C_α OOP motions
747	741	B_{1g}	C_α-N_α-C_α, Cu-N_α
768	746	E_g	C_β OOP motions, C_β-C_γ-H OOP
812	798<br>802	B_{2g}<br>B_{1g}	macroring def., C_δ-Cl, C_β-C_γ-H<br>isoindole def., Cu-N_α
832	856	A_{1g}	macroring breathing, C_β-C_γ-H, Cu-N_α
862	880	E_g	C_β-C_γ-H OOP
984	967	B_{2g}	C_α-N_β-C_α, N_α-Cu-N_α, C_γ-C_δ-C_δ, C_β-C_γ-C_δ, C_δ-Cl
1025	1074	B_{2g}	macroring def., N_α-Cu-N_α, C_δ-Cl
1050	1083	B_{1g}	C_β-C_γ-H, C_δ-Cl
1127	1153	A_{1g}	C_α-N_α-C_α, isoindole def., C_δ-Cl
1169	1181	B_{1g}	C_α-N_α-C_α, isoindole def., C_δ-Cl, C_β-C_γ-H
1188	1198	B_{2g}	C_α-N_β-C_α, C_β-C_γ-H, C_β-C_β-C_γ, C_α-N_α
1206	1214	B_{2g}	C_β-C_γ-H, C_α-C_β-C_β
1303	1302	B_{1g}	C_β-C_γ-H, C_γ-C_δ-Cl, C_α-N_α, C_α-C_β
1323	1310<br>1311	A_{1g}<br>B_{1g}	C_β-C_β, C_δ-C_δ, C_α-N_β-C_α, C_β-C_γ-H
1393	1381	A_{1g}	isoindole def., C_α-C_β, C_α-N_β-C_α, C_α-C_β
1405	1398	B_{2g}	C_β-C_γ-H, C_α-N_α, C_α-C_β, C_α-N_β-C_α, C_β-C_γ-H
1452	1435	B_{1g}	C_β-C_γ-H, C_β-C_γ, C_β-C_β
1478	1460	B_{2g}	C_α-N_β, C_α-C_β
1528	1560	B_{1g}	C_α-N_β, C_β-C_β
1578	1571<br>1573	A_{1g}<br>B_{1g}	C_β-C_β, C_δ-C_δ, C_α-N_β<br>C_α-N_β, C_β-C_β, C_δ-C_δ
1602	1603	B_{2g}	C_α-C_β, C_β-C_γ, C_γ-C_δ

Table S5. The experimental and calculated Raman and IR wavenumbers (cm^{-1}) and assignments of the most intense vibrations in the IR and Raman spectrum of $CuPcCl_{16}$.

Experimental wavenumbers		Calculated wavenumbers	Sym-metry	Assignment
Raman	IR			
219		222	B_2	N_α-Cu-N_α, C_β-C_γ-Cl, C_γ-C_δ-Cl, C_β-C_γ-Cl, C_γ-C_δ-Cl
229		228	B_1	C_γ-C_δ-Cl, C_γ-C_δ-Cl, N_α-Cu-N_α
236		233	A_1	C_β-C_γ-Cl, C_γ-C_δ-Cl
258		238	A_1	Cu-N_α, C_β-C_γ-Cl, C_α-N_β-C_α
286		328	A_1	Cu-N_α, C_β-C_γ-C_δ
327		334	E	benzene def., Cu-N_α, N_α-Cu-N_α
330		337	B_1	benzene def., Cu-N_α
343		338	A_1	C_β-C_γ-C_δ, Cu-N_α
364		368	B_2	C_γ, C_β and N_β OOP motions
375		373	E	C_γ, C_β and N_β OOP, Cu-N_α
439		487	B_1	isoindole def., C_α-N_β-C_α
504		509	A_1	macroring breathing, Cu-N_α
	509	503	E	macroring def., Cu-N_α,
542		538	E	isoindole def. OOP
590		583	A_1	C_γ and C_δ OOP motions
	597	592	E	C_γ and C_δ OOP motions
	606	599	E	C_α-C_β-C_γ, C_γ-Cl, Cu-N_α
638		631	B_1	C_γ-Cl, C_δ-Cl, C_α-C_β-C_γ
647	648	638	E	C_α-N_β-C_α, C_γ-Cl, C_δ-Cl
	669	657	E	C_γ-C_δ-Cl, C_β-C_γ-Cl, C_α-C_β-C_γ, C_α-N_β-C_α
680		671	A_1	C_α-N_β-C_α, macroring def.
696		695	B_1	C_α-C_β-C_γ, C_γ-Cl, isoindole def.
701		699	A_1	C_α-N_α-C_α, Cu-N_α, C_α-N_β-C_α
	712	714	E	C_α, N_α OOP motions
728		718	B_2	C_α, N_α, N_β OOP motions, C_α-C_β-C_γ
735		737	B_2	C_α-N_α-C_α, Cu-N_α, benzene def.
	747	747<br>748	E<br>E	C_α-C_β-C_β, C_α-N_α-C_α, C_γ-Cl<br>macroring def., C_δ-Cl, C_γ-Cl
768	769	761	B_2	C_α-N_α-C_α, Cu-N_α, benzene def., C_γ-Cl
800	779	767	E	C_α-N_α-C_α, Cu-N_α, benzene def., C_γ-Cl
818		801	A_1	macroring breathing, C_δ-Cl, C_γ-Cl
	823	812	E	macroring def, C_δ-Cl, C_γ-Cl
	897	935	E	macroring def, C_δ-Cl, C_γ-Cl
	948	938	B_2	isoindole def., C_γ-Cl, C_δ-Cl
952		946	A_1	C_α-N_α-C_α, Cu-N_α, C_β-C_γ-C_δ, C_γ-Cl
	966	948	E	C_α-N_β-C_α, isoindole def., C_γ-Cl
974		971	B_1	C_α-N_β-C_α, isoindole def., C_γ-Cl, C_δ-Cl
1078		1077	B_1	C_α-N_α, benzene def., C_δ-Cl, C_γ-Cl
	1095	1105	E	C_α-N_α, benzene def., C_δ-Cl, C_γ-Cl, C_α-N_α-C_α
	1152	1154	E	C_α-N_{α}·, C_β-C_γ-C_δ, C_α-N_β-C_α, benzene def.
1184		1180	A_1	benzene breathing, C_α-N_α, C_δ-Cl, C_γ-Cl
	1184	1193	E	benzene breathing, C_α-N_α, C_δ-Cl, C_γ-Cl
1196		1194	B_2	benzene breathing, C_δ-Cl, C_γ-Cl
1208		1220	B_1	C_α-N_α, isoindole def., C_δ-Cl, C_γ-Cl
	1210	1195	E	benzene breathing, C_δ-Cl, C_γ-Cl
	1276	1282	E	C_α-C_β, C_γ-C_δ, C_γ-C_δ-Cl

		1288	B_2	C_β-C_β, C_γ-C_δ, C_δ-C_δ, C_γ-C_δ-Cl
1277		1289	A_1	C_β-C_β, C_δ-C_δ, isoindole def.
1299		1293	B_2	C_α-N_α-C_α, C_α-N_α, C_α-C_β, C_δ-C_δ
	1305	1298	E	C_β-C_β, C_δ-C_δ, C_α-N_β, Pyrrole def.
1317		1307	A_1	C_β-C_γ, C_α-C_β-Cl, C_α-N_α, C_α-N_α-C_α
	1320	1324	E	C_α-N_α, C_α-N_α-C_α, C_α-C_β
1340	1366	1328	B_2	C_α-C_β, C_γ-C_δ, isoindole def.
1384		1385	A_1	C_α-N_α, C_α-N_α-C_α, C_β-C_β, isoindole def.
	1390	1378	E	C_α-C_β, C_γ-C_δ, isoindole def.
1419		1460	B_1	C_α-N_β, C_α-C_β, C_β-C_γ
	1450	1465	E	C_α-N_β, C_α-C_β, C_β-N_α-C_α
1477		1466	E	C_α-N_β, C_α-C_β
	1497	1511	E	C_α-N_β, C_α-N_α
1501		1508	A_1	C_α-N_β, $C_{\alpha'}$-N_β, $C_{\alpha'}$-$C_{\beta'}$, C_α-C_β
1537		1529	B_2	C_β-C_β, C_δ-C_δ, C_α-N_α
1540	1543	1533	E	C_α-$C_{\beta,}$ C_β-C_γ, C_γ-C_δ
1545		1539	B_1	C_α-$C_{\beta,}$ C_β-C_γ, C_γ-C_δ
1557	1558	1557	B_2	C_α-N_β

Table S6. Measured peak positions, intensities and assigned *hkl* indexes for the XRD pattern of $CuPcCl_{16}$.

**2θ (°, CuKa)**	**d (Å)**	**I/I_0 (%)**	**hkl**
6.127	14.43	100	110
6.807	12.99	35.5	020
10.155	8.711	9.4	200
12.245	7.228	16.1	220
13.609	6.507	12.0	040
15.608	5.678	15.5	310
17.002	5.215	13.8	240
18.386	4.826	14.8	330
21.499	4.133	4.1	420
22.936	3.878	14.4	350
24.569	3.623	15.5	440
25.077	3.551	4.2	-221 / -111
25.863	3.445	9.9	510
26.764	3.331	19.0	-421 / -131
27.390	3.256	14.0	021
28.513	3.131	10.3	370
30.890	2.895	28.4	131 / 550
31.407	2.848	15.8	190
32.984	2.716	2.0	221
33.740	2.657	4.9	061/ 640
34.420	2.606	11.7	480/ 0 10 0 /-371
36.232	2.479	7.2	710

Table S7. Measured peak positions, intensities and assigned *hkl* indexes for the XRD pattern of $CuPcCl_8$.

2θ (°, CuKa)	2θ [see Ref. 36]	d (Å)	I/I_0 (%)	hkl
6.093	6.1	14.51	100	100
6.601	6.6	13.39	23.1	010
8.428	8.5	10.49	45.2	1-10
9.499	9.5	9.311	30.7	110
12.209		7.250	4.4	021/1-1-1/200
13.886	14.0	6.378	68.7	001/1-20
14.544	14.5	6.091	50.8	121/210
16.927		5.238	2.0	2-20
18.375		4.828	5.4	2-3-1/300
18.874		4.702	3.9	201
19.922		4.457	2.9	030
21.430		4.147	2.1	041
22.151		4.013	3.1	2-30/2-11/3-3-1
23.949		3.716	5.4	320
24.897	24.9	3.576	22.4	1-21/1-1-2
25.459		3.499	1.8	12-1/2-4-2/4-2-1
26.206		3.401	10.9	410
26.541		3.358	9.2	241/4-20
27.164	27.1	3.283	39.2	1-5-1
28.061		3.180	5.8	140/2-40/3-4-2
28.815		3.098	1.5	330/401
29.386		3.039	7.7	242/420/421
29.754		3.003	2.9	152/4-4-1
32.474		2.757	4.7	
33.649		2.664	4.6	
34.336		2.612	4.8	
35.077		2.558	2.3	
